# Attitudes towards COVID-19 precautionary measures and willingness to work during an outbreak among medical students in Singapore: a mixed-methods study

**DOI:** 10.1186/s12909-021-02762-0

**Published:** 2021-06-04

**Authors:** Tricia Jia Wen Koh, Abel Ho Zhi Ling, Christine Li Ling Chiang, Gabriel Sheng Jie Lee, Hannah Si En Tay, Huso Yi

**Affiliations:** 1 Yong Loo Lin School of Medicine, National University of Singapore, Singapore, Singapore; 2 Saw Swee Hock School of Public Health, National University of Singapore and National University Health System, Singapore, Singapore

**Keywords:** COVID-19, Health policy, Bioethics, Health literacy, Attitude of health personnel, Medical education

## Abstract

**Background:**

The COVID-19 pandemic has revealed challenges that medical students face when healthcare systems are under intense pressure. There is a need to assess medical students’ education needs in pandemic preparedness. The objective of this mixed-methods study was threefold: (1) to assess COVID-19 perceived efficacy, susceptibility, and anxiety in relation to health literacy; (2) to describe attitudes towards a policy of precautionary measures against COVID-19 and willingness to work during an outbreak; and (3) to examine multilevel factors associated with willingness to work.

**Methods:**

An online survey was conducted among 263 medical students in Singapore during the lockdown period in July 2020. Participants were surveyed on COVID-19 related literacy, perceptions, anxiety, attitudes towards a policy of precautionary measures, and willingness to work during an outbreak. Bivariate and multivariate analyses were used to determine the factors associated with the key outcome variable of willingness to work. In addition, open-ended questions were used to assess medical education needs, which were reported using thematic analysis.

**Results:**

Perceived adequacy of COVID-19 information was associated with higher perceived efficacy, lower perceived susceptibility, and lower anxiety levels among the students. Medical students were mostly supportive of COVID-19 precautionary measures except for relatively intrusive measures like in-home surveillance. The degree of willingness to work during an outbreak varied based on certain conditions, in particular family’s health and safety, and was associated with self-efficacy, perceived susceptibility, and hospital capacity of outbreak management.

**Conclusions:**

Medical students’ attitudes towards a policy of precautionary measures varied depending on legality, financial and psychological support, and privacy concerns. Health literacy played an important role in increasing the efficacy of protection against COVID-19 and reducing pandemic-related anxiety among medical students. Their willingness to work during an outbreak was increased by an effective policy of precautionary measures, hospital capacity to manage a pandemic, and assurance of family safety. Medical education should include pandemic preparedness to better prepare students to aid in pandemics, with emphasis on public health policy and ethics coupled with clinical training targeted to managing outbreaks.

**Supplementary Information:**

The online version contains supplementary material available at 10.1186/s12909-021-02762-0.

## Background

Globally, healthcare systems’ ability and capacity to effectively respond to infectious disease outbreaks have been tested by the COVID-19 pandemic [1, 2]. One of the key challenges was healthcare personnel shortages. In regions with a high prevalence of COVID-19, medical students were either recruited, or they voluntarily participated in clinical care as frontline healthcare workers [3]. Without proper training, medical students in the frontline would feel unsure and incapable of performing their roles; they might experience stress and burnout more easily than trained clinicians [4–7]. This could cause unintentional harms in patient care and population health [8]. The current pandemic has underscored the importance of early education on pandemic preparedness in the medical education curriculum. This would train medical students to be equipped to meet challenges from emerging infectious disease outbreaks, pandemics, and other public health crises [9].

Pandemic preparedness is a multilevel concept evaluated in the following domains: (1) system resilience of resources for testing, treatment and personal protective equipment (PPE), (2) academic and clinical competency on logistical challenges specific to a pandemic, (3) awareness and resources available for maintenance of optimal mental health, and (4) individual ability to make difficult decisions with regards to ethical principles and policies of pandemic control and patient treatment [8]. Many medical schools do not include pandemic preparedness training in their current curricula [8–12]. Traditionally, medical schools teach students to acquire knowledge and skills in controlled clinical environments. While this is safe, such learning environments do not ensure students are prepared to function in more ‘high risk’ situations like outbreaks, pandemics and other disasters [8].

During the COVID-19 pandemic, the roles of medical students ranged from being fast-tracked to enter the workforce [13], augmenting non-COVID clinical care [3], volunteering in the community as contact tracers, to being a liaison to the public regarding COVID-19 practices [6]. Studies reported that many medical students felt unprepared [7, 9] or were actually unprepared in terms of the skills required to work in the pandemic [14, 15], with several reporting that their mental health was affected as well [16]. They lacked adequate knowledge on the use of PPE [14, 17, 18] due to insufficient training on PPE and infection control [18]. Medical students with a lack of relevant public health knowledge of COVID-19 misperceived the pandemic situation, reporting high levels of stress and anxiety which led to difficulty making appropriate medical judgments [19].

Knowledge of public health policy and ethics is an important component in pandemic preparedness. There is a duty to take precautionary measures to protect the public from justifiable dangers even under conditions of uncertainty [20]. While precautionary measures such as mobility restriction, quarantine, closure of public places and schools, and lockdowns are necessary for the control of the spread of infectious disease, the degree of restriction and intrusion of privacy requires ethical justifications [20]. Public health and clinical ethics competency enables medical students to understand the complexity of decision-making in health policy and patient care, and empowers student to communicate effectively with their family, community members and patients [21, 22]. Altogether, pandemic preparedness influences healthcare workers’ willingness to work. Access to adequate protection, perceived competence in essential clinical skills, and protection of family members were found to increase willingness to work during an outbreak [23–25]. Understanding multifactorial domains that determine willingness to work during an outbreak is crucial in developing effective pandemic preparedness of a nation [24].

In Singapore, a nationwide pandemic framework pandemic preparedness has been developed since the Severe Acute Respiratory Syndrome (SARS) outbreak in 2003 [26]. Healthcare systems underwent simulated pandemic scenario training to assess and improve their outbreak protocols [27]. The National Medical Undergraduate Curriculum Committee outlines the Outcomes and Standards for Undergraduate Medical Education in Singapore and includes appropriate use of personal protective equipment and infection control in relation to procedures [28]. The residency programmes incorporated pandemic response into their training and used this pandemic as an opportunity to impart skills on adapting to unpredictable conditions [29, 30]. However, educational needs assessment in pandemic preparedness from the perspective of medical students has not been conducted.

In the current study, we assessed knowledge, source of information, perceived literacy, efficacy and susceptibility of COVID-19, attitudes towards a policy of precautionary measures, willingness to work during an outbreak, and anxiety relating to COVID-19 among medical students. The objective of the study was threefold: (1) to assess COVID-19 perceived efficacy, susceptibility, and anxiety in relation to health literacy of the pandemic; (2) to describe attitudes towards precautionary measures and willingness to work based on various conditions; and (3) to examine factors associated with willingness to work during an outbreak. In addition, the qualitative analysis of open-ended questions aimed to detail the educational needs of medical students and complement quantitative findings.

## Methods

### Sampling and participants

An online survey was conducted among preclinical and clinical year students in a medical school in Singapore from 2 to 15 July 2020 during the “circuit breaker”, the local term for lockdown. The undergraduate medical programme is a five-year course leading to the degrees of Bachelor of Medicine and Bachelor of Surgery (MBBS) with approximately 300 students being enrolled each year. The survey invitation, as a form of informed consent, was sent via a link to all medical students through each batch’s Telegram group, an online communication platform. The recruitment approach was approved by the medical school education office. A reminder was sent during the second recruitment. A total of 263 students participated in the survey. The survey questions were reviewed by the medical school, and the study was approved by the university research ethics committee (IRB/DERC SSHSPH-032).

### Measures

In addition to social demographic information, academic year and clinical exposures, the following domains were assessed in the survey. The survey questionnaire is included as an (Additional file 1).

Knowledge and perception of COVID-19 included participants’ exposure to COVID-19, their knowledge on symptoms, viral transmission and incubation, basic epidemiology, disease course, and associated risks in different age groups. The source of information on COVID-19 was assessed by preferred sources, the types of information sources participants relied on most, receipt of unsolicited (“fake”) information, and perceived COVID-19 information adequacy (“whether you feel that you have enough information on COVID-19”). Perceived efficacy and perceived risk of contracting COVID-19 were assessed using the question of perceived competency of protecting oneself and others if infected, perceived susceptibility, and worries about contracting COVID-19.

Attitudes towards a policy of precautionary measures were assessed with nine questions using a 5-point Likert scale (1 = strongly disagree to 5 = strongly agree). The items were adapted from a scale that was previously validated in a study of attitudes towards quarantine policy following the SARS outbreak [31]. The original scale had 15 items that addressed issues of legality of restrictive measures, perceived effectiveness of quarantine, and financial and psychological supports that should be provided for those affected by quarantine orders with four factors of justification, sanctions, burdens, and safeguards. We selected nine items and modified the item descriptions specific to the study sample of medical students and local COVID-19 policy. The final items were reviewed by experts in bioethics, psychometrics, and medical education, and included (1) the government’s authority to issue quarantine orders, (2) no exception of quarantine, (3) legal penalties if a quarantine order was broken, (4) imprisonment, (5) use of GPS wristband to monitor, (6) in-home surveillance camera to monitor, (7) provision of free food and lodging, (8) provision of free counselling services for those in quarantine if needed, and (9) financial compensation for absence at work due to quarantine. The scale was reliable in the study sample (*α* = .71).

Willingness to work and reasons for it were developed based on the review of empirical studies that assessed willingness to work during infectious disease outbreaks [25] and reasons for willingness to respond to public health crises [32]. The measure of willingness to work was assessed based on the following scenario using a 5-point Likert scale (1 = least willing to 5 = most willing): (1) if fatality rate of infection is lower than 10%, (2) if fatality rate is greater than 10%, (3) if adequate PPE is not available, (4) if effective vaccine or prophylaxis is not available, and (5) if an alternative living arrangement is not provided to minimise the risk of infection to family. The scale was highly reliable in the study sample (α = .83). Then, participants responded to “how important are the following reasons in consideration of your willingness to work in response to an infectious disease outbreak?”: (1) personal safety and health, (2) family safety and health, (3) hospital capacity, (4) hospital preparedness, (5) stigma against people exposed, and (6) government support for the family.

COVID-19 related anxiety was assessed by modified questions of the General Anxiety Disorder Assessment (GAD-7). We added “due to COVID-19” at the end of each item in the GAD-7: (1) feel nervous (2) worry, (3) worry too much, (4) trouble relaxing, (5) restless, (6) easily annoyed, and (6) feel afraid as if something awful might happen. Response options were “not at all,” “several days,” “more than half the week,” and “nearly every day,” scored as 0, 1, 2, and 3, respectively. The scale was highly reliable in the study sample (*α* = .94).

#### Qualitative assessment

In addition, the survey included open-ended questions to better understand the education or training needed for medical students to be adequately prepared to assist on the frontline.

### Analysis

Descriptive statistics were used to describe the demographics of participants and their responses to attitudes towards quarantine policies and willingness to work during an outbreak. T-tests were conducted to compare the scores on perceived efficacy and susceptibility of COVID-19 and COVID-19 related anxiety by health literacy on the disease. Spearman correlations between the variables were conducted to check multicollinearity. Multiple regression analyses were conducted to examine the factors associated with willingness to work. The regression model was adjusted for demographic variables significantly associated with the outcome variable at the bivariate level. Backward elimination was used in multivariate regression analysis to identify independent predictors of the outcome variable in the model. For qualitative data, we used thematic analysis [33]. Two authors first read the responses thoroughly, then each conducted open coding independently and produced preliminary codebooks with themes. The two codebooks were checked by the authors and consolidated with the last author, who was an expert in qualitative data analysis. The final coding was made with the consensus of all coders, and categorisation was made.

## Results

### Characteristics of participants

Of 263 survey participants, the mean age was 21.9 years old with an equal proportion of men (*n* = 123, 47%) and women (*n* = 140, 53%), and the majority were Chinese (*n* = 232, 88%). About three quarters (*n* = 205, 78%) were in their clinical training years, with half (*n* = 153, 58%) in their first year of clinical training. The months of clinical exposure ranged from 1 to 55 months (M = 3.5 + 6.72). The vast majority (over 95%) of participants had good knowledge of factual information regarding COVID-19 - its clinical presentation, mode of transmission, and measures on controlling its spread. However, there was variability in participants’ understanding of susceptibility to COVID-19 across age groups. About one-third responded that older adults were more susceptible to COVID-19 infection compared with other age groups. With regards to the acquisition of COVID-19 information, 87% (*n* = 229/263) actively searched for information and their top three preferred sources were the government (*n* = 187, 71%), online (*n* = 140, 53%), and healthcare professionals (*n* = 95, 36%). About two-thirds of them (*n* = 144/229, 63%) felt they had sufficient information about COVID-19.

### COVID-19 literacy, perception, and anxiety

Table 1 shows perceived efficacy and susceptibility of COVID-19 and anxiety related to COVID-19 by the individual’s perceived information adequacy, which was assessed by ‘perception of having sufficient information.’ Compared to those without perceived information adequacy, students with perceived information adequacy reported higher efficacy of protecting themselves from COVID-19 infection (t = 4.90, *p* <  0.001) and preventing the transmission of COVID-19 to others (t = 3.36, *p* <  0.001), lower perceived susceptibility (“worry about infection in the past month”, t = − 2.06, *p* <  0.05), less worry about COVID-19 infection in the future (t = − 2.95, *p* <  0.01), and a lower score regarding COVID-19 anxiety (t = − 2.90, *p* <  0.001).

**Table 1:** Efficacy, perceived susceptibility and COVID-19 anxiety by perceived COVID-19 information adequacy

	Enough COVID-19 information		t-test
	Yes (62.9%)	No (37.1%)	
	M + SD	M + SD	
How confident are you that you can protect yourself against COVID-19?	3.77 + 0.84	3.23 + 0.75	4.90***
If you became infected with COVID-19, how confident are you that you could avoid spreading the virus to others?	3.48 + 1.17	2.94 + 1.19	3.36***
If you were to develop symptoms of COVID-19 tomorrow, how worried would you be?	3.83 + 1.12	3.90 + 1.01	− 0.49
In the past one month, how worried have you been about catching COVID-19?	2.27 + 1.08	2.57 + 1.08	− 2.06*
How likely do you think it is that you will contract COVID-19 over the next 1 month?	2.13 + 0.83	2.31 + 0.62	−1.76
How much do you worry that your family and friends could get infected with COVID-19?	3.33 + 1.17	3.79 + 1.05	−2.95**
Anxiety related to COVID-19	2.45 + 3.87	4.15 + 4.76	− 2.90**

* *p* < 0.05 ** *p* < 0.01 *** *p* < 0.001

### Attitudes towards a policy of COVID-19 precautionary measures

**Fig. 1:**
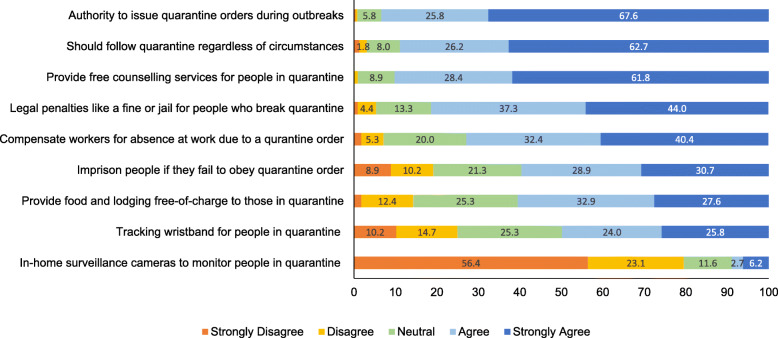
Attitudes towards policy of precautionary measures of COVID-19

A total of 225 students responded to all the items on the scale. Students were generally agreeable towards COVID-19 precautionary measures, although this varied according to how strict and invasive each measure was (Fig. 1). The majority of students agreed that authorities should have the ability to issue quarantine orders during outbreaks (*n* = 210, 93%); if someone is issued a quarantine order by the government, they should follow it regardless of circumstances at work or home (*n* = 200, 89%); the government should provide free counselling to those in quarantine (*n* = 204, 90%); legal penalties should be dealt to people who break quarantine orders (*n* = 183, 81%), and the government should financially compensate workers for absence at work due to a quarantine order (*n* = 164, 73%). Meanwhile, about half agreed that people should be imprisoned should they disobey quarantine orders (*n* = 134, 60%); the government should provide food and lodging free-of-charge to those being quarantined (*n* = 136, 61%); and GPS tracking wristbands should be given to people in quarantine (*n* = 112, 50%). For the abovementioned three items, about a quarter remained neutral, and the rest disagreed. For the measure of close monitoring using CCTV, 80% (*n* = 179) disagreed that in-home surveillance cameras could be used to monitor people in quarantine, and 9% (*n* = 20) agreed.

### Willingness to work during an outbreak

**Fig. 2:**
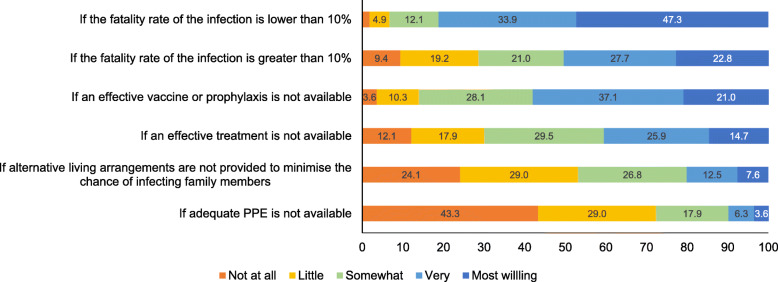
Willingness to Work during an Infectious Disease Outbreak

A total of 224 students responded to all the items on the scales of willingness to work and reasons for it. There was a variation in students’ response to their willingness to work, depending on different hypothetical situations regarding COVID-19 (Fig. 2). Most students (*n* = 182, 81%) were willing to work if the fatality rate was less than 10%. However, only half (*n* = 113, 51%) were willing to work if the fatality rate was more than 10%, with 29% (*n* = 64) unwilling to work. About half of the students (*n* = 130, 58%) were willing to work even if no effective vaccine or prophylaxis was available, and comparatively, less than half (*n* = 91, 41%) were willing to work if no effective treatment was available. Only 20% (*n* = 45) were willing to work if alternate living arrangements were not provided to minimise chances of infecting their family members, and 10% (*n* = 22) were willing to work if adequate PPE is not available.

### Reasons for willingness to work

**Fig. 3:**
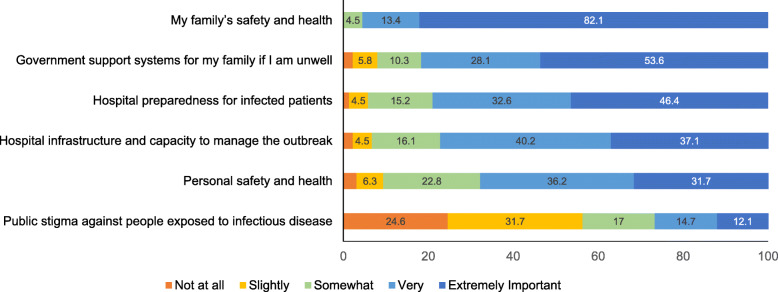
Reasons for Willingness to Work during an Infectious Disease Outbreak

Family’s safety was reported as the main factor for considering their willingness to work during an infectious disease outbreak (Fig. 3). Majority responded that their family’s safety and health were very important when considering working during an outbreak (*n* = 214, 96%). Government support for their family if they fell sick (*n* = 183, 82%) was very important in influencing their decision to work as well. Following the concern about family safety, hospital preparedness for infected patients (*n* = 177, 79%) and hospital infrastructure and capacity to manage the outbreak (*n* = 173, 77%) were important reasons for willingness to work. This was followed by personal safety and health (*n* = 152, 68%). Public stigma against people exposed to infectious diseases (*n* = 60, 27%) was the least important reason affecting their willingness to work.

### Regression analysis of willingness to work

Table 2 shows the findings of univariate and multivariate regression analyses of willingness to work. It was associated with perceived efficacy and susceptibility of COVID-19, attitudes towards the government’s authority in setting quarantine measures, having no exception in compliance of quarantine, provision of psychological service for COVID-19, reasons of family’s safety and health, hospital capacity to manage the outbreak and anxiety related to COVID-19. In the multiple regression model, the following four variables were found to be significantly associated with willingness to work: perceived efficacy and susceptibility of COVID-19 (B = 0.280, SE = 0.073, *p* <  0.001 and B = -0.159, SE = 0.056, *p* = 0.005, respectively), attitudes towards no exception in compliance of quarantine (B = 0.155, SE = 0.073, *p* = 0.035), and hospital capacity to manage outbreak (B = 0.124, SE = 0.062, *p* = 0.048).

**Table 2:** Regression of willingness to work: univariate and multivariate model

	Univariate			Multivariate		
	B (SE)	β	*p* value	B (SE)	β	*p* value
Self-efficacy of COVID-19 protection	0.363 (0.071)	0.323	< 0.001	0.280 (0.073)	0.249	< 0.001
Perceived susceptibility of COVID-19	−0.205 (0.057)	− 0.235	< 0.001	− 0.159 (0.056)	− 0.181	0.005
Authority of quarantine measures	0.278 (0.095)	0.193	0.004			
No exception in compliance of quarantine	0.171 (0.077)	0.147	0.028	0.155 (0.073)	0.134	0.035
Provision of psychological service for COVID-19	0.205 (0.092)	0.148	0.027			
My family’s safety and health	0.375 (0.122)	0.201	0.002			
Hospital capacity to manage outbreak	0.160 (0.066)	0.16	0.017	0.124 (0.062)	0.124	0.048
Psychological distress related to COVID-19	−0.032 (0.015)	−0.145	0.031			

### Qualitative findings

The scheme of categories and subthemes of medical education needs is presented in Table 3.

**Table 3:** Thematic scheme of COVID-19 medical education needs

Category	Themes	Representative Quotes
Protocol Improvement	• Hospital workflow and protocols	“More knowledge on protocols taken in hospitals during an infectious disease outbreak”
	• Roles and risks of student involved	“What medical students can or cannot do on the frontlines – specifics of role and risks involved”
	• Protection of family from COVID-19	‘Would like to know what additional precautions I should be taking at home to avoid infecting my family”
	• Knowledge tailored to needs	“COVID-19 disease crash course lecture or rounds on current information on COVID”
Skill Enhancement	• Personal protective equipment (PPE)	“Regular PPE training. Some basic procedures since Year 1 so we can assist in the case of an outbreak”
	• Real-life simulation and experiences	“More hands-on practice. How different practicing on a dummy/mannequin is compared to real life persons.”
	o COVID-specific clinical exposure	“Proper practice on investigation and management procedures for COVID-19 suspect cases”
	o Refresher courses	“More training sessions to refresh memory”
	o Post-exposure protocol	“Steps to be taken after contact with suspect case”
	o Increased clinical exposure	“More real-life experience”
	o Management of emergencies	“Symptomatic management as well as how to spot emergencies that should be escalated immediately”
	o Patient interaction	“The emotional aspect/being sensitive to patients”

### Protocol improvement

#### Hospital workflow and protocol

As a member of the patient care team in hospitals, medical students follow and assist in the daily duties of the medical team. Participants wished to have “more knowledge on protocols taken in hospitals during an infectious disease outbreak” so that they could be informed about a “proper workflow of the frontline area they work in”. This would allow students to be better prepared to contribute and avoid compromising the safety of the medical team and the patient during the pandemic. Students encountered differences in workflows between regional health clusters and the institutions they belonged to. Therefore, the protocols need to be more institution-specific and clearly distinguish the differences across the various healthcare settings. The infectious disease control protocol students requested for includes “updates on related preventive measures.”

#### Roles and risks of medical student involved

Some students were confused about their roles in frontline care and were uncertain about their level of involvement during COVID-19. They responded that there is a need for their roles as medical students to be clearly defined and requested that the guidelines explain “what medical students can or cannot do on the frontline” and “different roles and job scopes.” They also expressed a need to be aware of the “specifics of roles and risks involved.” This could be on an institutional level, such as being updated on the “number of cases the hospital is dealing with” or on the basis of individual procedures such as the risk of exposure to COVID-19 while performing swabs.

#### Protection of family from COVID-19

Consistent with the quantitative findings, students deemed their family’s health and safety as an important factor in influencing their willingness to work. To prevent the potential spread of COVID-19 to family members, they need to be assured about infection control at the various stages of their day to day posting experience such as “preparations before coming to the wards,” “how to sanitise themselves before leaving the workplace and after reaching home”. Beyond learning from seniors, they wished to have it in the form of a written protocol such as an “information sheet on family protection between home and hospital”. Other suggestions included an arrangement for “alternative accommodation”, such as dormitories if students are not willing to place vulnerable family members at risk. Additionally, students suggested that the protocol would need to include post-exposure procedures, such as “steps to be taken after contact with the suspect case,” and “how to manage in the event of PPE breach” to adequately protect their family should they work in the frontline.

#### Knowledge tailored to needs

Students wished to be more informed about COVID-19. As suggested by the quantitative findings, their poor literacy of COVID-19 affected perceived efficacy and susceptibility and psychological distress. Thus, in the face of difficulties in navigating the deluge of new information released about the disease, students wanted information to be standardised and approved by trusted faculty members. This would help to prevent misunderstandings and ensure uniform competency, alleviating students’ distress and increasing their confidence. Suggestions included a “lecture on current information” with information about the “symptomology in chronological order” and an “update of scientific investigations and health policy and management”.

### Skills enhancement

#### Regular PPE training

Students wished to have regular training on donning and doffing of PPE. As one student said:

*Regular PPE training is important so that we do not forget what we learn. It may be beneficial to include training of some basic healthcare procedures from the start of Year 1 so that we can assist in the case of a pandemic. It was helpful to have a re-fitting of masks during the outbreak so that we are adequately prepared since some people’s mask sizes might have changed.*

Such training needed to be “constant, repeated and updated with respect to the latest infectious disease outbreak.” While PPE training had already been included in the curriculum, students were concerned that current amounts of training were inadequate. Especially in this pandemic with a highly contagious disease, students understood the importance of appropriate PPE techniques to protect themselves from infection. Regular PPE training throughout medical school years could increase both the confidence and willingness of students to respond to an outbreak and assist on the frontline.

#### Real-life simulations and experiences

There was a need for hands-on practice of fundamental clinical procedures, especially for infectious outbreaks such as nasopharyngeal swabbing, phlebotomy, and post-exposure protocol. One student commented on the difference between simulations and real clinical experience:

*I need to have more hands-on practice for swabbing and phlebotomy and move on from practising on mannequins to proper training in clinical settings. I am not sure how different it is practising on a dummy/mannequin compared to real-life patients.*

Students responded that “real-life simulation before actual exposure to patients” would increase their confidence and that the current pandemic could provide them with an opportunity for “more real-life experiences.” For example, many students expressed that they would like to have “proper practice on investigation and management procedures for COVID-19 suspect cases.” Such skills were not routinely taught in the normal curriculum as they are specific and context dependent. Students can learn better when exposed to real-life scenarios where they could practice decision-making under high-stress environments. This practice could include more training on patient interaction, as suggested by participants, in terms of “how to work with uncooperative patients and the emotional aspect of being sensitive to patients.” Students also identified the need for there to be “more training sessions to refresh memory”, as one student remarked:

*I would simply like to be clear of the procedures in the event of a confirmed case and also what I need to do if I am suspected of contracting COVID-19. Refreshers for the techniques required would also be appreciated.*

This reinforces the importance of having repeated and regular clinical training. Students shared that insufficient clinical exposure was a gap in their training. A student shared that:

*I think it would be good if the school allowed us to voluntarily go to the dormitories or other community settings to perform swabs. However, I do understand that it is an environment that puts us, students, at high risk of contracting COVID-19.*

The need for more clinical exposure did not only refer to COVID-19 specific exposure. Students wished to learn “symptomatic management as well as how to spot emergencies that should be escalated immediately”. More training to deal with general emergencies and exposure to high-risk environments could prepare them to assist in situations like the current pandemic.

## Discussion

The study highlighted the importance of literacy of public health policies and clinical aspects of COVID-19 in increasing perceived efficacy and decreasing susceptibility and anxiety among medical students. A range of views towards a policy of precautionary measures and willingness to work during an outbreak was also explored. Importantly, the study documented individual knowledge, attitudinal and healthcare system factors that affected willingness to work at multiple levels. Our qualitative findings of educational needs enriched the survey findings and contextualised medical students’ needs in Singapore’s current situation. With increasing urbanisation, widespread accessibility of travel and worsening climate change contributing to the increase in the incidence of infectious diseases [34], the arrival of the next major pandemic appears to be a matter of “when” not “if”. Early exposure and more thorough incorporation of health literacy of the present pandemic will better prepare the medical workforce for future pandemics [35].

Knowledge of COVID-19 and preventive practices were widely diffused with legal enforcement in the community over the course of the pandemic, especially during the time of the lockdown period. In the study, while all students reported accurate knowledge and the majority actively sought information through various sources, fewer felt that their knowledge was sufficient. This suggests that medical students need more specific COVID-19 knowledge that tailors to their needs and career. Without such knowledge, they felt susceptible and anxious about getting infected by the virus.

The finding that students held varying levels of support towards various policies employed to control the pandemic suggests a need for ethical reasoning to be included in the teaching of public health ethics. Given the highly contagious nature of the virus, their support for quarantine measures and enforcement was strong. However, policies with greater restrictions on personal freedom – GPS tracker – and potential intrusion for privacy – in-home CCTV surveillance – received lower levels of support from students. The lower rates of acceptance were in line with the public’s hesitancy of a wearable token for GPS contact tracing in the country [36]. Medical students are primarily trained to respect patients’ autonomy and confidentiality according to medical ethics principles. Public health response to control infectious disease outbreak might be seen as contradictory. There is a need for greater education on public health ethics in medical schools. This would enable medical students to play an important role in participating in, collaborating with, and advocating for public health policy, especially in the face of a complex new media environment saturated with misinformation [37]. With a better understanding of public health policies and medical practices, they can serve as good role models for the general public [38].

It is important to note that multiple factors were considered in determining willingness to work, not only individual competency but also appraisal of hospital capacity and pandemic response systems. In particular, the protection of family is crucial in the decision-making of willingness to work, and this finding was also supported by our qualitative assessment of education needs. Previous studies also reported the role of family protection in determining willingness to work among medical professionals [25]. This notion of balancing family safety and health with the duty to care should be addressed in pandemic preparedness policy and curriculum. Willingness to work is critical in the enhancement of coordinated pandemic preparedness at the national level.

By integrating quantitative and qualitative findings, specific recommendations for medical curricula are described. Pandemic preparedness in medical education should encompass the following topics: knowledge, clinical training, public health policies and ethics, and resilience training [37]. Knowledge should include disaster medicine training (such as how to manage logistical challenges in pandemics, infection control principles, etc.), and up-to-date information about the disease [5, 9, 39–41]. Clinical training should cover two areas: PPE training in real-world scenarios for self-protection [17], and increased clinical exposure to infectious disease settings under proper supervision to reduce their chance of contracting or transmitting the disease [40]. Public health and clinical ethics applied in infectious disease outbreaks and pandemics should be included in the curriculum, such as the principles and ethical considerations of precautionary measures taken to control outbreaks and healthcare workers’ duty to care even in times of outbreaks [42, 43]. Resilience training pre-pandemic should focus on individual’s confidence by being supported by their workplace to protect themselves and family members, coping methods including individual resilience strategies such as psychological first aid and organisational resilience in the form of supportive relationships, and self-efficacy in handling an outbreak [44]. This would help medical students deal with the stress associated with working in such high-risk environments.

### Limitations

There are several limitations to this study. Its cross-sectional nature limits inference on the directionality of associations. The study was conducted using an online survey form during the lockdown. About a quarter of the total number of medical students in the school responded to the survey. There might be non-response bias as students with less interest in COVID-19 did not participate in the study, and students who were busy with frontline practices might not have time for survey participation. Students with strong views or opinions on the subject would respond to the open-ended sections, while the views of the silent majority were not considered. This could cause misrepresentation of the medical student population at large. However, this did not invalidate the responses received, as they highlighted problems reflected in other studies, and also contribute to acknowledging what is lacking in today’s medical education. When the study was conducted in July 2020, we did not expect that COVID-19 pandemic would be sustained for such a long time. Future research needs to examine the effect of the prolonged pandemic on burnout and willingness to work among those working in healthcare sectors.

## Conclusion

The COVID-19 pandemic has underscored the importance of pandemic preparedness in the medical education curriculum to equip medical students with knowledge and skills to better fulfil their roles in a pandemic. Increased literacy of public health policy, ethics and medical practices improves competency and resilience in times of pandemics. Family safety and health should be addressed to promote medical students’ willingness to work. Medical education should empower medical students to embrace new competencies suited to addressing re-emerging pandemics. This can be achieved through improving health literacy of the pandemic, clinical training, greater incorporation of public health policy and ethics education, and resilience training.

## Supplementary Information

**Additional file 1.**

## Data Availability

The datasets used and/or analysed during the current study are available from the corresponding author on reasonable request.

## Abbreviations

COVID-19: Coronavirus disease 2019
PPE: Personal protective equipment
