# Supplementary material for: Attitudes towards COVID-19 precautionary measures and willingness to work during an outbreak among medical students in Singapore: a mixed-methods study
Source: BMC Med Educ. 2021 Jun 4;21:317. doi: 10.1186/s12909-021-02762-0 (PMC8176668; doi:10.1186/s12909-021-02762-0)
Supplement: Supplementary file 1 — Additional file 1. [file 12909_2021_2762_MOESM1_ESM.pdf]

Knowledge, Attitudes & Concerns About COVID-19 among NUS YLLSoM Medical Students

We are a team of NUS medical students. We are conducting a survey to understand attitudes and concerns about preventive measures taken to stop the spread of COVID-19. This is an anonymous survey. You will not be required to provide any names or information that can be used to identify you. All survey information will be kept confidential. Your participation is voluntary. You can withdraw from the survey at any time without penalty. We appreciate your honest responses. This should take no longer than 20 minutes of your time. Thank you for your time and contribution. If you consent to participate in the survey, please click the button, ‘start’ to begin.

Demographic Information

1. Age:
2. Gender: M/F
3. Ethnicity:
4. Year of Study: M1/M2/M3/M4/M5
5. Number of months of clinical exposure: ____ months

Knowledge of COVID-19

1. Do you personally know anyone infected with COVID-19? Yes ___ No ___
   If YES, who are they (check all that apply)
   a. Family
   b. Friends
   c. Co-workers (seniors)
   d. Healthcare workers
   e. Others (specify)

2. What are the symptoms of COVID-19? (check all that apply)
   a. Fever, Cough, Shortness of breath
   b. Diarrhoea
   c. Loss of sense of taste/smell
   d. Muscle ache
   e. Sore throat
   f. Runny nose

3. Do you think COVID-19 can be spread by?
   a. Coughing/sneezing & close personal contact (e.g. handshake, hugs)
   b. Touching contaminated surfaces (eg: door handles)
   c. Touching/eating raw meat
   d. Touching/eating contaminated food
   e. Mosquito bites

4. On average, how long do you think it takes for a person to develop symptoms if he/she is infected with COVID-19?
   a. 1-3 days
   b. 4-6 days
   c. 7-14 days
   d. Over 2 weeks

5. Can an asymptomatic person infected with COVID-19 transmit the virus to others? Yes ___ No ___

6. Which of the following do you think is the important steps in controlling the spread of COVID-19? (check all that apply)
    a. Isolation and identification of suspect cases
    b. Wearing masks in public
    c. Daily temperature monitoring
    d. Temperature screening at malls, airports and other shared public spaces
    e. Staying at home as much as possible

7. Which of the following are considered as suspect cases? (select all that apply)
    a. A patient who has cough and breathlessness and who has recently come back from Europe
    b. A patient who has a mild cough and whose neighbour is a confirmed case
    c. A patient who has cough, runny nose and sore throat without travel or contact history
    d. An asthmatic patient who has breathlessness without travel or contact history

8. Which of the following are considered as ‘close contacts’? (select all that apply)
    a. one who passed by a confirmed case in the corridor
    b. one living in the same place as a confirmed case
    c. one who provided care for the patient and had close physical contact (within 2m) with the patient
    d. one who had close physical contact with someone issued a Quarantine Order (QO)

9. What percent of individuals infected with COVID-19 experience a fatal disease course in Singapore?
    a. <1%
    b. 2-3%
    c. 10%
    d. 15%

10. Comparing between 3 groups of the population, elderly (>65 years), adults (35-65 years) and the youth (12-35 years), who has the higher likelihood of the followings: (4 options for each part: elderly, adults, youth, same likelihood across all)

	Elderly	Adults	Youth	Same
10.1. Contracting COVID-19				
10.2. Transmitting COVID-19				
10.3 Developing severe disease and death from COVID-19				

Source of Information on COVID-19

1. Have you actively searched for information on COVID-19? Yes ___ No ___

2. Which is your preferred source of information regarding information about COVID-19? **Please choose top three sources.**
    a. Official government sources (e.g. MOH, HPB, SGSecure, Gov.sg broadcasts etc.)
    b. Newspapers
    c. Television (e.g. CNA, Channel 5/8/U, Vasantham, Suria)
    d. Internet search (e.g. Google, Yahoo, MSN, Bing)
    e. Social media (e.g. Facebook, Twitter, Instagram)
    f. Healthcare professionals
    g. Family members and friends (e.g. Via Whatsapp/Telegram/WeChat/LINE/KakaoTalk)

3. Have you received any unsolicited information about COVID-19 from these social media sources that you did not deliberately search for? (these include news stories, health advice, advertisements, personal stories)
    a. Facebook
    b. Whatsapp/WeChat/KakaoTalk, etc.
    c. Twitter
    d. Instagram

4. Do you feel that you have enough information about COVID-19? Yes ___ No ___

5. Is there additional information about COVID-19 you feel you need? If so, please specify

Protection from COVID-19

Please indicate your opinion, 1 = not at all, 3 = neutral, and 5 = very much

	Not At All ------------------------- Very Much				
1. How confident are you that you can protect yourself against COVID-19?	1	2	3	4	5
2. If you became infected with COVID-19, how confident are you that you could avoid spreading the virus to others?					
3. If you were to develop symptoms of COVID-19 tomorrow, how worried would you be?					
4. In the past one month, how worried have you been about catching COVID-19?					
5. How likely do you think it is that you will contract COVID-19 over the next 1 month?					
6. How much do you worry that your family and friends could get infected with COVID-19?					

Attitudes Towards Quarantine

Please indicate your opinion, 1 = strongly disagree, 3 = neutral, and 5 = strongly agree

	Strong Disagree ------------- Strongly Agree				
1. MOH should have the authority to issue quarantine orders to suspect cases during mass outbreaks of infectious diseases	1	2	3	4	5
2. If someone is issued a quarantine order by MOH, they should follow it regardless of their circumstances at work or at home	1	2	3	4	5
3. People who break quarantine orders should face stiff legal penalties like a fine or jail.	1	2	3	4	5
4. The government should be able to imprison people if they fail to obey quarantine orders.	1	2	3	4	5
5. MOH should use a tracking wristband for people who are serving quarantine orders.	1	2	3	4	5
6. MOH should use in-home surveillance cameras to monitor people serving quarantine orders.	1	2	3	4	5
7. The Government should provide food and lodging free-of-charge to those in quarantine.	1	2	3	4	5

8. The Government should provide free professional counselling services so that people serving quarantine orders have adequate access to psychological support if needed.	1	2	3	4	5
9. The government should financially compensate workers for absence at work due to a quarantine order.	1	2	3	4	5

Willingness to Work

1. Rate your willingness to perform medical duties during infectious disease outbreak.

I, as a medical student, am willing to perform my duties (e.g., nasopharyngeal swabbing, phlebotomy), given the following conditions

	Least Willing --------------------- Most Willing				
1.1. If the fatality rate of the infection is lower than 10%	1	2	3	4	5
1.2. If the fatality rate of the infection is greater than 10%					
1.3. If adequate PPE was not available					
1.4. If an effective vaccine or prophylaxis is not available					
1.5. If an effective treatment is not available					
1.6. If alternative living arrangements were not provided to minimise the chance of infecting family members					

2. How important are the following reasons in the consideration of your willingness to work in responses to infectious disease outbreak?

	Least Important ------------- Most Important				
2.1 Personal safety and health	1	2	3	4	5
2.2. My family's safety and health	1	2	3	4	5
2.3. Hospital infrastructure and capacity to manage the outbreak	1	2	3	4	5
2.4. Hospital preparedness for infected patients	1	2	3	4	5
2.5. Public stigma against people exposed to infectious disease	1	2	3	4	5
2.6. Government support systems for my family if I am unwell	1	2	3	4	5

Generalized Anxiety Disorder 7-item (GAD-7)

Before and during the circuit breaker period (7th April - 1st June), how often have you been bothered by any of the following problems on average per week?

1. Feeling nervous, anxious or on edge as a result of Covid-19?

	Not at All	Several days	Over half the days	Nearly every day
1.1. Before CB				
1.2. During CB				

2. Not being able to stop or control worrying about Covid-19?

	Not at All	Several days	Over half the days	Nearly every day

2.1. Before CB				
2.2. During CB				

3. Worrying too much about COVID-19 related issues

	Not at All	Several days	Over half the days	Nearly every day
3.1. Before CB				
3.2. During CB				

4. Trouble relaxing as a result of Covid-19?

	Not at All	Several days	Over half the days	Nearly every day
4.1. Before CB				
4.2. During CB				

5. Being so restless that it is hard to sit still as a result of Covid-19?

	Not at All	Several days	Over half the days	Nearly every day
5.1. Before CB				
5.2. During CB				

6. Becoming easily annoyed or irritable as a result of Covid-19?

	Not at All	Several days	Over half the days	Nearly every day
6.1. Before CB				
6.2. During CB				

7. Feeling afraid as if something awful might happen as a result of Covid-19?

	Not at All	Several days	Over half the days	Nearly every day
7.1. Before CB				
7.2. During CB				

8. If you have experienced psychological distress due to COVID, have you talked to someone else about it? (check all that apply)
   a. Family
   b. Friends
   c. Professional help
   d. I did not talk to anyone about it

Needs Assessment

1. Have you been PPE-trained? Yes ___ No ___

2. Do you know your N95 mask size? Yes ___ No ____

3. How confident are you in donning PPE in the right sequence?

   (not confident at all) 1-----2-----3-----4-----5 (very confident)

4. If you were to don full PPE (Gloves, Respirator, Gown, Goggles), what would the correct order of removal be, in order to prevent exposure to contaminants?
    a. Mask > Gown > Gloves > Eye protection
    b. Mask > Eye protection > Gloves > Gown
    c. Gloves > Mask > Eye protection > Gown
    d. Gloves > Gown > Eye protection > Mask
    e. Unsure

5. Do you remember the 7 steps of hand washing? Yes ___ No ___

6. How confident are you in performing the 7 steps correctly?
    (not confident at all) 1-----2-----3-----4-----5 (very confident)

7. How confident are you in preventing yourself from getting infected in the wards?
    (not confident at all) 1-----2-----3-----4-----5 (very confident)

8. How confident are you in your supervisor's instructions to protect you from COVID-19 in the wards?
    (not confident at all) 1-----2-----3-----4-----5 (very confident)

9. Based on your experience, what additional training/knowledge from YLLSoM would you require in order to be adequately prepared to assist on the frontline? (open-ended)
